# Mucilicious methods: Navigating the tools developed to Arabidopsis Seed Coat Mucilage analysis

**DOI:** 10.1016/j.tcsw.2024.100134

**Published:** 2024-12-11

**Authors:** Susana Saez-Aguayo, Dayan Sanhueza, Vicente Jara, Benjamin Galleguillos, Alfonso Gonzalo de la Rubia, Asier Largo-Gosens, Adrian Moreno

**Affiliations:** a Centro de Biotecnología Vegetal, Laboratorio Mucilab, Facultad de Ciencias de la Vida, Universidad Andrés Bello, Santiago 8370146, Chile; b ANID- Anillo de Investigación en Ciencia y Tecnología - Chilean Fruits Cell Wall Components as Biotechnological Resources (CHICOBIO) ACT210025, Talca, Chile; c ANID - Millennium Science Initiative Program - Millennium Nucleus for the Development of Super Adaptable Plants (MN-SAP), Santiago, Chile; d Área de Fisiología Vegetal, Universidad de León, 24071, León, España, Spain

**Keywords:** Mucilage, pectin, cellulose, hemicellulose, methods

## Abstract

During the last decades, Arabidopsis seed mucilage has been extensively studied to gain insight into the metabolism of pectin, hemicellulose and cellulose. This review aims to provide a comprehensive examination of the techniques used to understand the composition and structure of *Arabidopsis* mucilage. Moreover, we present novel findings from mucilage analysis, including the separation of pectic domains within the mucilage, offering a fresh perspective on utilizing traditional techniques to analyze mucilage mutant lines.

## 1 Introduction

The plant cell is surrounded by a complex and dynamic structure known as the cell wall, which plays a crucial role in various processes throughout plant growth, including cell elongation, plant defense, and stress tolerance (Anderson and Kieber, 2020). Plants have two distinct types of cell walls: the primary and the secondary cell walls, each with a unique composition influencing their stiffness and function (Anderson and Kieber, 2020). In young cells, the primary cell wall — located between the middle lamellae and cell membrane — facilitates cell elongation and differentiation due its flexibility and porosity. In contrast, the secondary cell wall is synthesized in specialized cells within the vascular system, fibers and other sclerenchymatous cells (Delmer et al., 2024). This layer deposited between the primary cell wall and the cell membrane after the cell has finished its expansion, is more rigid and less porous (Meents et al., 2018; Delmer et al., 2024). The primary cell wall is primarily composed of cellulose, pectins, hemicelluloses, and proteins, while the secondary cell wall consists mainly of cellulose, hemicelluloses and lignin (Delmer et al., 2024). The composition and proportion of each polysaccharide, as well as their interactions, vary based on the plant species, tissue, and cell type, making it challenging to understand the intricate role of the cell wall in regulating diverse physiological processes (Anderson and Kieber, 2020; Cosgrove, 2022; Geitmann and Bacic, 2024; Delmer et al., 2024).

Due to the complexity and numerous interactions among cell wall polysaccharides, most current methods for analyzing plant cell walls are destructive, requiring polysaccharides hydrolysis, which often results in the loss of structural information, and spatial distribution of each component within the wall (Alonso Baez and Bacete, 2023). This limitation has driven the need for simpler cell wall models to help elucidate the synthesis and organization of cell wall structures. Approximately 20 years ago, Arabidopsis seed coat mucilage was characterized for the first time (Western et al., 2001), and has since become a model system for studying polysaccharides synthesis, modification, and organization (Voiniciuc et al., 2015a; Šola and Dean, 2019).

**Fig. 1:**
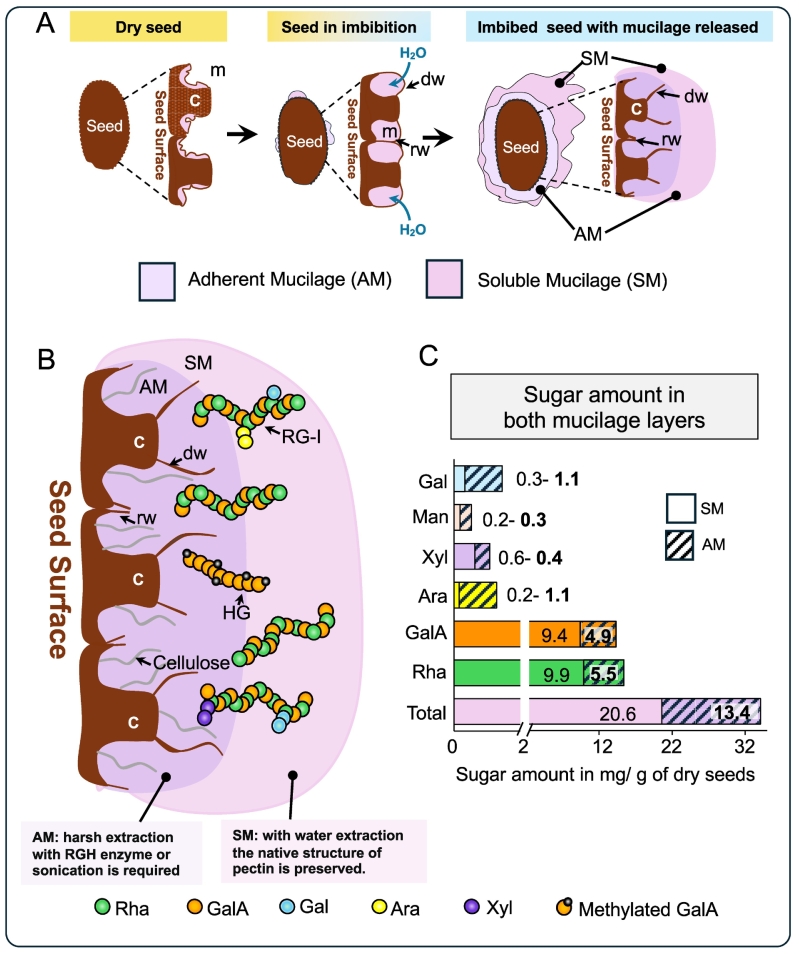
Structure and composition of Arabidopsis seed coat mucilage. A. Mucilage release from mature dry seeds. This panel illustrates the process of mucilage release, providing an overview of the seed coat epidermal cells. m, mucilage; c, columella rw, radial wall; dw, distal wall: SM, soluble mucilage, AM, Adherent mucilage. B. Composition of Arabidopsis mucilage. Arabidopsis mucilage primarily consists of pectins, specifically RG-I and HG. These pectins are anchored to the seed surface through interactions with RG-I xylan-cellulose fibers. Minor components, such as galactoglucomannans, AGPs, and RG-II are not represented here. c, columella rw, radial wall; dw, distal wall: SM, soluble mucilage, AM, Adherent mucilage. C. Pectin sugar content mucilage layers. This panel shows the average sugar content (expressed in mg/g of dry seeds) in both mucilage layers. The values are averaged from data obtained in studies by Macquet et al., 2007a, Macquet et al., 2007b, Saez-Aguayo et al. (2013), Fabrissin et al. (2019), and Parra-Rojas et al. (2023).

The mucilage in Arabidopsis seed coat is a gel-like structure produced by specialized epidermal cells called mucilage secretory cells (MSCs) between 6 and 12 days after pollination (DAP) (Western et al., 2004). This process encompasses several well-described phases: synthesis, deposition, maturation, and desiccation of mucilage, all occurring within MSCs (Saez-Aguayo et al., 2014; Francoz et al., 2015; Saez-Aguayo and Largo-Gosens, 2022; Xu et al., 2023). Upon hydration of mature dried seeds, the polysaccharides present in the mucilage expands, exerting pressure on the MSC radial walls (Fig. 1 A). This pressure causes rupture of the radial cell wall, releasing both soluble and adherent mucilage layers, which together form the characteristic Arabidopsis seed coat mucilage (Fig. 1 A; Arsovski et al., 2009; Saez-Aguayo et al., 2014; Saez-Aguayo and Largo-Gosens, 2022).

Arabidopsis mucilage contains all the major groups of polysaccharides groups found in plant cell walls, predominantly pectins (90–95 %) along with smaller amounts of additional components. The mucilage primarily consists of rhamnogalacturonan-I (RG-I), with lesser quantities of homogalacturonan (HG), cellulose, galactoglucomannans, xylans, xyloglucan, and arabinogalactan proteins (AGPs) (Fig. 1B and C; Macquet et al., 2007a; Saez-Aguayo et al., 2013; Voiniciuc et al., 2015a; Shi et al., 2017). Although rhamnogalacturonan-II has been suggested to play a role in Arabidopsis mucilage organization, its presence within the mucilage layers remains unclear (Shi et al., 2017).

## 2 Arabidopsis mucilage: Unravelling structure, release, and composition

### 2.1 A Sticky mucilage: a structured gel-like structure primarily composed of rhamnogalacturonan-I and homogalacturonan

Arabidopsis mucilage, though jelly-like structure, is highly organized and consists of two distinct layers: Soluble Mucilage (SM) and Adherent Mucilage (AM) (Fig. 1 A; Western et al., 2000; Macquet et al., 2007a, Voiniciuc et al., 2015a). As indicated by its names, the SM layer is readily extracted by incubating mature seeds in water; gentle shaking removes most of the SM, exposing the AM layer, which remains tightly bound to the seed coat integument (Fig. 1 A). Both layers share a similar composition, primarily rich in rhamnogalacturonan I (RG-I); however, the AM contains higher levels of other components, such as HG, cellulose, and hemicellulose (Fig. 1B and 1C; Macquet et al., 2007a, Macquet et al., 2007b; Voiniciuc et al., 2015a).

### 2.2 The predominant RG-I pectic domain found in mucilage represents a structure that has yet to be fully elucidated

The SM fraction is a water-soluble, primarily RG-I- based layer (Macquet et al., 2007a; Voiniciuc et al., 2015a), with the advantage of being extractable without harsh treatments, allowing direct access to the native rheological structure of its polysaccharides (Macquet et al., 2007a; Williams et al., 2020). This feature represents a notable advantage in studying cell wall components, as many traditional methods are destructive and may lead to the loss of critical information about the polymer's native structure and ionic interactions within the matrix.

RG-I in mucilage is characterized as a flexible polysaccharide within a backbone of galacturonic acid (GalA) linked to rhamnose (Rha) and sparse side-chains of arabinan, galactan, or arabinogalactan (Fig. 1B; Macquet et al., 2007a, Macquet et al., 2007b; Saez-Aguayo and Largo-Gosens, 2022). In wild-type Arabidopsis Col-0 seeds, RG-I from SM has an average mass of 600 kDa, consisting of approximately 1845 [Rha-GalA] dimers subunits, which represents an exceptionally long polymer (Macquet et al., 2007a; Sullivan et al., 2011; Williams et al., 2020; Fabrissin et al., 2019; Saez-Aguayo et al., 2021). Notably, while most RG-I in SM has this 600 kDa mass, a small fraction (about 10 %) has a very high molecular weight (> 40,000 kDa), corresponding to self-assembled RG-I structures similar to cellulose or amyloid polymers (Macquet et al., 2007a; Williams et al., 2020). RG-I synthesis occurs in the Golgi apparatus and is mediated by enzymes such as: rhamnosyltransferase 1 (RRT1) and the recently identified rhamnogalacturonan galacturonosyltransferase 1 (MUCI70/RGGAT1) (Takenaka et al., 2018; Amos et al., 2022). Additionally, galacturonosyltransferase-like 5 (GALT5) has been implicated in RG-I formation, acting as a terminator of RG-I polymer length (Kong et al., 2013). It is proposed that RG-I is initially synthesized with arabinan and galactan side-chains, which are subsequently degraded during mucilage maturation by α-arabinofuranosidases like BXL1, and β-galactosidases like MUM/BGAL6, yielding a smooth RG-I polymer (Dean et al., 2007; Macquet et al., 2007b; Arsovski et al., 2009; Williams et al., 2020). However, the glycosyltranferases responsible for synthesizing these side-chains of mucilage have not yet been identified.

### 2.3 Cellulose-Xylan involved in pectin anchoring to the seed surface

The adherence of the AM layer to the seed surface is driven by pectin attachment to cellulose microfibrils (Harpaz-Saad et al., 2011; Mendu et al., 2011; Sullivan et al., 2011; Griffiths et al., 2014, Griffiths et al., 2015 and Griffiths et al., 2016). Unlike pectins and hemicellulose, synthesized in the Golgi apparatus, cellulose microfibrils are produced at the plasma membrane by cellulose synthase complexes (CSCs) —membrane-bound protein complexes formed by cellulose synthases (CESAs) and other associated proteins (Sullivan et al., 2011; Griffiths et al., 2015). The intermolecular interactions between cellulose and pectin in composite hydrogels occur only when pectin is present during cellulose synthesis and depend on its degree of methylesterification (Lopez-Sanchez et al., 2017).

In mucilage, *CESA1*, *CESA2*, *CESA3*, *CESA5*, *CESA9* and *CESA10* are thought to be involved in cellulose biosynthesis (Griffiths and North, 2017). Among these, CESA3 and CESA5 are key for cellulose synthesis in mucilage and the formation of cellulosic ray, while CESA2 and CESA9 contribute to radial wall thickening. Although *CESA1* and *CESA10* are likely candidates based on their expression during mucilage synthesis, their roles remains to be demonstrated (Sullivan et al., 2011; Mendu et al., 2011; Griffiths et al., 2015). Live-cell imaging of GFP-CESA movement upon hydration strongly suggests that cellulose is deposited in a coil-like structure around the cytoplasmic column by CSCs, unwinding during mucilage extrusion to form the cellulose ray in MSCs (Griffiths et al., 2015; Griffiths and North, 2017). Mutants affecting cellulose synthesis in MSCs, such as *cesa3*, *cesa5*, and *cesa10*, exhibit altered cellulose assembly, leading to complete solubilization of the AM layer in water (Harpaz-Saad et al., 2011; Mendu et al., 2011; Sullivan et al., 2011; Griffiths et al., 2015). Similar AM adherence loss occurs in *mum5–1* mutants whith xylosyltransferase mutations, which display a redistribution of pectin from the AM to the soluble layer. Linkage analysis of the RG-I-enriched-fraction in *mum5–1* reveals reduced xylan and cellulose linkage compared to wild-type, underscoring that xylan linked to RG-I is essential for mucilage adherence to the seed surface (Ralet et al., 2016; Voiniciuc et al., 2018). While the precise positioning of these RG-I side chains remains unclear, recent studies on *urgt246* and *muci70* mutants show that xylan content correlates with RG-I polymer production, emphasizing xylan's importance for mucilage structure and seed adhesion (Fabrissin et al., 2019; Saez-Aguayo et al., 2021).

### 2.4 Homogalacturonan plays a substantial role in mucilage structure and release

Homogalacturonan (HG) is the second main pectin domain in mucilage, comprising approximately 5 % of the total non-cellulosic polysaccharides in mucilage (about 0.9 mg/g of dry seed; Fig. 1B and C Saez-Aguayo et al., 2013). HG consists of a backbone of galacturonic acid, which can be methylesterified (Willats et al., 2001; Macquet et al., 2007a). Synthesized in the Golgi apparatus with high methylation, HG is subsequently secreted into the apoplast, where it is demethylesterified by pectin methylesterases (PMEs), which catalyze the release of methanol from the carboxyl group of GalA (Wormit and Usadel, 2018; Levesque-Tremblay et al., 2015). PME activity is, in turn, regulated by specific proteins called PME inhibitors (PMEIs). In Arabidopsis mucilage, HG has a low degree of methylesterification (DM) at around 8.6 %, indicating precise regulation during mucilage formation (Saez-Aguayo et al., 2013; Turbant et al., 2016). Upon seed imbibition, immunolabeling reveals a structured pattern of HG within the AM, with higher DM in the outer AM and more demethylated HG in the inner AM (Macquet et al., 2007a, Macquet et al., 2007b, Saez-Aguayo et al., 2013; Turbant et al., 2016; Parra-Rojas et al., 2023, Shi et al., 2018). Studies on Arabidopsis mucilage have identified specific PMEs (e.g., HMS and PME58) and PMEIs (e.g., PMEI6, PMEI13, PMEI14, PMEI15, PMEI6), that further validate the utility of this model (Table 3). Notably, the strong phenotype of *pmei6* mutants, which exhibit delayed mucilage release and a lack of epidermal cell wall fragmentation (Saez-Aguayo et al., 2013)*,* raises questions about the origin of HG domains in the AM. Researchers suggest that HG may migrate through the mucilage matrix upon seed imbibition, possibly originating from distal cell wall breakage.

**Fig. 2:**
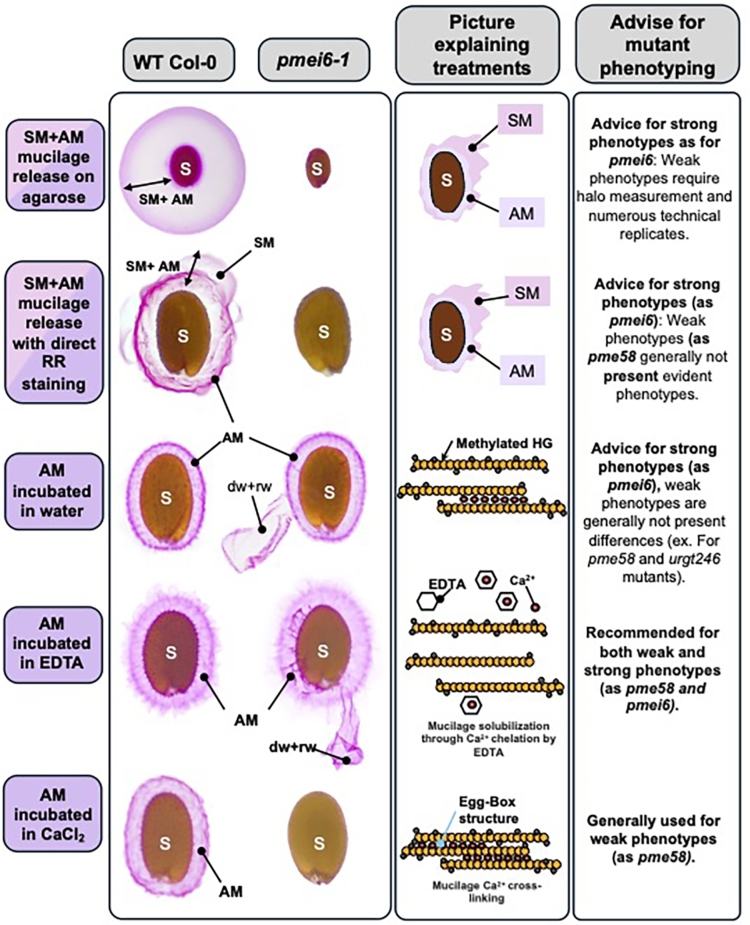
Comparison of *pmei6–1* mutant phenotype with WT Col-0 using Ruthenium Red staining across five different procedures. Seeds were sown on stained agarose and subjected to direct imbibition with RR, which reveals released mucilage from both mucilage layers. Incubation with different solvents (water, EDTA, CaCl2), removes soluble mucilage, offering insights into the AM structure and potential changes in HG methylesterification and/or mucilage density. Scale bar = 100 μm.

Regardless of its precise origin, subtle modifications in HG methylesterification significantly impact mucilage release, as shown in studies of the *pmei6–1* mutant (Fig. 2; Saez-Aguayo et al., 2013). Moreover, research has shown that PMEI6 interacts with PER36 (a class-III peroxidase), forming microdomains in the cell wall that establish optimal properties for effective mucilage release (Saez-Aguayo et al., 2013, Kunieda et al., 2013, Francoz et al., 2015). Additionally, *O*-acetyl esterification, another key feature of HG structure, is under investigation. Although the specific acetyltransferase remains unconfirmed, studies suggests that trichome birefringence-like (TBL) family proteins may play a role in *O*-acetylation (Shahin et al., 2023). Recent work on TBL38, and its role alongside PMEI6 and PER36, has underscored the significance of HG acetylation in ensuring proper mucilage release (Dauphin et al., 2023). Future research is needed to elucidate the balance between HG methylation and acetylation in Arabidopsis mucilage.

### 2.5 Galactoglucomannans and arabinogalactan proteins: Possible roles in mucilage production

In mucilage, galactoglucomannan (GGM) follows a structural pattern of repeating disaccharide [4)-β-Glc-(1,4)-β-Man-(1] (Voiniciuc et al., 2015c; Yu et al., 2018). Despite being a minor component (approximately 5 % of total sugars) in mucilage, GGM content is higher here than in other tissues. The structure of GGM in mucilage is influenced by glycosyltransferases such as CSLA2 (Liepman et al., 2005) and MAGT1/MUCI10 (Voiniciuc et al., 2015c), along with GDP-Man pyrophosphorylase VTC1 and its regulator KJC1, influence (Conklin et al., 1999; Nishigaki et al., 2021). Despite insights into GGM structure, its interactions with other mucilage polysaccharides remain unclear (Yu et al., 2018; Nishigaki et al., 2021).

Similarly, arabinogalactan proteins (AGPs) are minor components of mucilage. They consist of a core-protein backbone O-glycosylated by complex carbohydrates, mainly galactose and arabinose, with variable length and domain complexity, and often feature arabinogalactan (AG) type II side chains and a glycosylphosphatidylinositol (GPI) lipid anchor (Sommer-Knudsen et al., 1998). AGPs have been observed forming direct links with pectins and hemicelluloses, creating a complex network (Tan et al., 2023). Despite limited information exists on AGP roles in mucilage, studies indicate that SOS5 and FEI2 are part of a pathway responsible for synthesizing seed coat mucilage, which includes pectin and cellulose (Harpaz-Saad et al., 2011). Additionally, three AGP glucuronosyltransferases (GLCATa, GLCATb, GLCATc) and two AGP galacturonosyltransferases (GALT2, GALT5) have been implicated in seed coat mucilage synthesis (Basu et al., 2016; Ajayi et al., 2021; Zhang et al., 2021); however, their mutants exhibited only mild phenotypes, affecting mucilage solubility and decreasing AM adherence (Basu et al., 2016; Ajayi et al., 2021; Zhang et al., 2021).

Recent work by Tan et al. (2023 and 2024) indicates that AGPs are associated with pectins and, particularly in siliques, with HG. This observation raises questions about AGP involvement in mucilage structure: are AGP linked to HG or to other components? Regardless of the specific linkages, AGPs appear to play a role in organizing pectin components around cellulose microfibrils, an arrangement essential for cellulose ray formation and mucilage adherence to the seed surface. For instance, disruptions in glycosyltransferase family 14 members, responsible for adding Me-GlcA to AG glycans, led to loss of adherent mucilage, altered cellulose ray formation, and changes in seed coat morphology (Ajayi et al., 2021). Variation in AGPs content can be effectively detected in adherent and soluble mucilage layers using anti-AGPs-antibodies in dot-blot assays (Griffiths et al., 2016; Parra-Rojas et al., 2023).

## 3 Exploring Common Cytological Methods for Phenotyping Mucilage Release and Composition

### 3.1 Methods for assessing mucilage release

Arabidopsis mucilage is rapidly released upon seed imbibition, making it possible to asses structural mutations by quantifying the total mucilage release (Macquet et al., 2007a; Voiniciuc et al., 2015a). A rapid method for evaluating total mucilage release is to sow seeds on 0.05 % agarose, with or without Ruthenium Red (RR) staining, or by directly immersing mature seeds in a RR staining solution (typically 0.005 % to 0.02 % *w*/*v*) (Fig. 2; Western et al., 2004; McFarlane et al., 2014; Voiniciuc et al., 2015c). This method is well-suited to identify strong mucilage phenotypes, such as defects in mucilage release or loss of mucilage adherence. For detecting subtler phenotypes, we recommend sowing mature seeds on agarose plates and measuring the halo of mucilage release, while accounting for seed size (Fig. 2) (Miart et al., 2018; Parra-Rojas et al., 2023). Though this approach is slower, it enables more specific screening of subtle mucilage release phenotypes (Macquet et al., 2007a; Saez-Aguayo et al., 2017; Parra-Rojas et al., 2023). Another technique to quantify mucilage release involves creating a kinetic profile of mucilage extrusion in RR through the visualization over several minutes of the appearance of the mucilage halo, as described by Arsovski et al. (2009), Saez-Aguayo et al., 2013 and, Parra-Rojas et al. (2023).

### 3.2 Quantification of structural changes in adherent mucilage

To visualize changes in the AM structure, RR staining can be applied by shaking mature seeds in water to remove the SM and subsequently staining the AM (Macquet et al., 2007a, Macquet et al., 2007b). Enhanced visualization of AM structural changes with RR staining can be achieved by incubation with EDTA or CaCl_2_ (Fig. 2) to relax or densify the AM layer. EDTA chelates the calcium ions, relaxing the mucilage matrix by disrupting egg-box structures formed by demethylesterified HGs (Levesque-Tremblay et al., 2015). In contrast, CaCl_2_ favors the formation of these egg-box structures, densifying the AM (Decreux and Messiaen, 2005; Van Rooyen et al., 2023). These imbibition techniques help describe subtle changes in mucilage structure, typically associated linked to HG methylesterification alterations, as described in the *pmei6* mutant line (Fig. 2) (Saez-Aguayo et al., 2013, Saez-Aguayo et al., 2017; Turbant et al., 2016; Shi et al., 2018).

A more precise technique than RR staining for detecting AM structure changes involves whole-mount assay of AM from mature seeds using antibodies specific to pectin epitopes present in mucilage. To detect changes in HG structure, specific antibodies such as 2F4, JIM5, LM19, JIM7, and LM20 are commonly utilized, as these recognize egg-box structures and HG regions with varying methylesterification degrees and patterns (Table S1; Macquet et al., 2007a, Macquet et al., 2007b; Saez-Aguayo et al., 2013, Saez-Aguayo et al., 2017 and Saez-Aguayo et al., 2021; Parra-Rojas et al., 2023). For detecting RG-I, antibodies such as INRA-RU1, INRA-RU2, and CCRC-M36 are effective (Table S1; Ralet et al., 2010; Pattathil et al., 2010; Voiniciuc et al., 2015a; Sun et al., 2019). Although detecting RG-I lateral chains is challenging, it may be feasible using LM5 (galactan) and LM6 (arabinan) antibodies (Table S1; Macquet et al., 2007a; Saez-Aguayo et al., 2017). For HC components in mucilage, one can take advantage of the fact that LM25 recognizes three forms of xyloglucans (Pedersen et al., 2012); LM21 labels hetero-mannans (Voiniciuc et al., 2018); and CCRC-M139 and INRA-AXI detect xylan in AM (Table S1; Ralet et al., 2016; Voiniciuc et al., 2015b, Fabrissin et al., 2019). Though these antibody signals can be quantified, changes in labeling patterns often provide more information than strict quantification.

To visualize cellulose in mucilage, fluorescent dyes such as Calcofluor White and Scarlet Fast Red (S4B or Direct red) are effective (Harpaz-Saad et al., 2011; Griffiths et al., 2015). Changes in the amorphous and crystalline cellulose can be observed using CBM28 and CBM3a, respectively. To assess AM density, a method involving Dextran-FITC penetration into the mucilage capsule is valuable (Voiniciuc et al., 2018; Saez-Aguayo et al., 2021). Dextran-FITC, a fluorescently tagged molecule, reveals structural changes in mucilage matrix polysaccharide. It can be diluted in water, EDTA, or CaCl_2_ to study these changes and quantified using ImageJ (Schindelin et al., 2012). This approach is particularly useful for phenotyping mutants with subtle structural variations (Saez-Aguayo et al., 2021).

### 3.3 Assessment of seed epidermal cell wall phenotype

During seed imbibition, mucilage hydrates and expands, exerting pressure on the distal cell wall. This pressure leads to cell wall rupture and mucilage extrusion (Fig. 1 A). Epidermal cells typically break at the corners where the distal and radial cell walls intersect. Certain mutations can affect the stiffness of these cell walls, altering their susceptibility to breakage. To characterize this phenotype, scanning electron microscopy (SEM) is effective for determining the shape and size of the seed coat epidermal cells and columellae in dry mature seeds (Murovec et al., 2012; Wang et al., 2019; North et al., 2014; Voiniciuc et al., 2018; Parra-Rojas et al., 2023). Additionally, confocal microscopy, utilizing stains such as Calcofluor or Direct Red 23 to visualize cellulose, can reveal changes in size and shape resulting from altered mucilage release and distal cell wall breakage. This approach has been used to study several mutants, including *gosamt* and *cesa5* mutant lines (Sullivan et al., 2011; Voiniciuc et al., 2018; Parra-Rojas et al., 2023).

## 4 Biochemical analysis to evaluate sugar composition in both mucilage layers

### 4.1 The influence of extraction methods, acid hydrolysis, and usual analytical methods on monosaccharide composition

Methods for mucilage extraction have evolved significantly. Water has proven effective for extracting SM, as shown by Macquet et al. (2007a), and traditional pectin extraction with ammonium oxalate did not show substantial differences in SM extraction compared to water in Col-0 lines (Macquet et al., 2007a). It is estimated that SM contains approximately 20 mg of non-cellulosic sugars, primarily GalA and Rha (Fig. 1C). While the compositions of AM and SM are similar, AM has higher levels of Gal, Ara, and mannose, whereas SM contains more Xyl and cellulose (Fig. 1B). The proportion of sugars in SM is generally higher; but variations can occur based on plant cultivation conditions during seed maturation (Saez-Aguayo et al., 2021).

Compared to SM, extracting AM is more challenging due to its properties; AM cannot be easily separated from the seed and requires harsher extraction methods or specific enzymatic actions. In 2007, Macquet et al. developed a method using Rhamnogalacturonan hydrolase (RGH) to extract AM by breaking down smooth RG-I attached to the cellulose fibers of the seed surface. Other enzymes, such as endopolygalacturonase (endoPG) or cellulases, were less effective than RGH (Macquet et al., 2007a). Despite its efficacy, RGH is not commercially available. To overcome this limitation, Zhao and Qiao (2017) developed a sonication-based method for AM extraction, which is simpler but harsh, potentially causing contamination of sugars from seed surface into the mucilage. Table 1 summarizes AM sugar content extracted using RGH digestion (Fabrissin et al., 2019; Saez-Aguayo et al., 2021) and sonication (Parra-Rojas et al., 2019; Parra-Rojas et al., 2023).

**Table 1:** **Comparisons of sugars measured in the adherent mucilage with enzymatic digestion and sonication.** The table shows the concentrations of sugars forming pectins in AM, represented in mg/g of dry seeds and as percentage. The sonication method yields higher sugar extraction from the AM layer. Data were sourced from Fabrissin et al., 2019; Parra-Rojas et al., 2019 and Parra-Rojas et al., 2023; Saez-Aguayo et al., 2021.

	**ENZYMATIC DIGESTION**				**SONICATION**			
	Saez-Aguayo et al., 2021		Fabrissin et al., 2019		Parra-Rojas et al., 2019		Parra-Rojas et al., 2023	
***Sugars***	mg g-1 seed	%	mg g-1 seed	%	mg g-1 seed	%	mg g-1 seed	%
GalA	5.62	44.11	4.85	53.31	5.36	39.97	6.42	46.35
Rha	4.14	32.5	3.75	41.99	6.06	45.19	4.62	33.36
Ara	0.25	1.96	0.03	0.34	0.14	1.04	0.42	3.03
Xyl	0.14	1.1	ND	0	0.62	4.62	0.57	4.12
Man	0.29	2.28	ND	0	0.23	1.72	0.26	1.88
Gal	2.3	18.05	0.3	3.36	1	7.46	1.56	11.26
**Total sugars (AM)**	**12.74**		**8.93**		**13.41**		**13.85**	

The main sugars identified include GalA, Rha, and Glc, though variablity among studies, complicates direct comparison. Sonication generally, yielded higher levels of total sugars, total mucilage, and AM specifically. For individual monosaccharides, sonication resulted in a 29.7 % increase in total sugars compared to AM obtained without sonication (Table 1). Different methodologies did not show significant variation in the detection range of GalA; however, Rha levels varied from 3.75 to 6.06 mg/g of seed. A similar trend is observed for Gal, with enzymatic digestion yielding amounts between 0.3 and 2.3 mg, while sonication results ranged from 1 to 1.56 mg per gram of seed (Table 1). Glucose data was excluded from Table 1 due to substantial variability, which would skew the relative sugar proportions. With sonication, it is probable that distal and radial cell walls components were inadvertently included in the AM, possibly explaining the higher sugar content. Extraction duration could also influence the detected AM sugar content; for instance Saez-Aguayo et al. (2021) extracted AM by incubating seeds with RGH at 40 °C overnight. Uronic acids were quantified using the *m*-hydroxydiphenyl method, whereas neutral sugars were analyzed as alditol acetate derivatives by gas-liquid chromatography after hydrolysis with 2M trifluoroacetic acid at 121 °C for 2.5 h. Fabrissin et al. (2019) reported a decrease in total monosaccharides by over 50 %, possibly due to reduced incubation time with RGH, from overnight to 1.5 h (Fabrissin et al., 2019; Saez-Aguayo et al., 2021).

### 4.2 Impact of diverse hydrolysis and detection methods on the quantification of monosaccharide composition in mucilage layers

In addition to differences between mucilage extractions methods, quantifying monosaccharides in each layer involves hydrolyzing polysaccharides to break the linkages between sugars. After hydrolysis, individual acidic and neutral sugar are typically quantified using High-Performance Anion-Exchange Chromatography with pulsed amperometric detector (HPAEC-PAD) or Gas Chromatography-Mass Spectrometry (GC–MS). Trifluoroacetic acid (TFA) hydrolysis is often the preferred method due to its rapid reaction kinetics and volatility, which eliminates the need for neutralization (Fengel and Wegener, 1979). Typically, TFA is used at a concentration of 2 M and heated to 121 °C, with hydrolysis times varying across studies from 30 min to 2 h. It is important to note that TFA effectively cleaves pectin and hemicellulose polysaccharides while leaving cellulose unaffected. However, the monosaccharide yield can vary significantly depending on structural modifications and interactions among polysaccharides (Macquet et al., 2007a; Voiniciuc et al., 2015b and c; Saez-Aguayo et al., 2013, Saez-Aguayo et al., 2017, Saez-Aguayo et al., 2021). To eliminate non-cellulosic glucose, amylase may be added post-hydrolysis. In mucilage samples, cellulose can be recovered through centrifugation before proceeding with hydrolysis (Zhao and Qiao, 2017).

### 4.3 Colorimetric assays to determine the methanol, Galacturonic Acid and Rhamnose content in both mucilage layers

#### 4.3.1 Colorimetric assay for quantification of acidic and neutral sugars

Besides HPAEC-PAD and GC–MS, other techniques like colorimetric methods can be used to measure GalA and Rha content in mucilage layers. These colorimetric techniques, such as *m*-hydroxybiphenyl and orcinol assays (Blumenkrantz and Asboe-Hansen, 1973; Tollier and Robin, 1979), are currently employed for quantifying GalA and total sugars, providing an overview of the primary mucilage components. Cost-effective and rapid, these methods facilitate high-throughput screening of mucilage phenotypes, especially advantageous for small sample sizes typical of dry seeds. Colorimetric methods for GalA measurement are not only cheaper and faster but also require minimal sample quantities. When comparing GalA levels detected via colorimetric assays to HPAEC, colorimetric techniques often yield higher GalA values. This difference occurs because colorimetric methods detect GalA independently of polymer breakdown, unlike HPAEC, which requires full monomerization of GalA and may underestimate GalA content, potentially affecting the Rha/GalA ratio during mutant phenotyping. To improve accuracy, some studies combine neutral sugar quantification through TFA hydrolysis with colorimetric assays for GalA (Macquet et al., 2007a; Saez-Aguayo et al., 2013; Ralet et al., 2016; Saez-Aguayo et al., 2021). For Rha quantification, the orcinol method is similarly inexpensive and fast, though it detects both neutral and acidic sugars with different specificities. Additionally, colorimetric assays can be effective for kinetic studies on mucilage release, as they allow determination of the Vmax of mucilage release, quantified as GalA, in mutants like *pmei6* (Saez-Aguayo et al., 2013; Berger et al., 2021).

#### 4.3.2 Methanol determination using colorimetric assay

To assess the methanol content of HG domains, a commonly used method involves a colorimetric assay. This assay includes saponifying pectins with 0.2 M NaOH for 1 h (optimized for mucilage), after which the reaction is halted by neutralizing with hydrochloric acid (HCl). The methanol content is then determined using alcohol oxidase activity, following the protocol described in Saez-Aguayo et al. (2013). However, this method has challenges, as methanol's volatility necessitates conducting the process at lower temperatures to prevent evaporation. Additionally, determining the degree of methylation requires calculating the methanol-to- GalA ratio. This calculation is complicated because GalA content includes contributions from both RG-I and HG, which can significantly underestimate the actual HG methylation degree in the mucilage. For greater accuracy, isolating HG is essential. Saez-Aguayo et al. (2013) employed ethanol precipitation of HG after digesting RG-I, but alternative methods are needed given that RG hydrolases are currently not commercially available. Another alternative is to measure mucilage methylation using HPLC methods, as decribed in Levigne et al., 2002.

#### 4.3.3 Other methods for investigations of mucilage structure

HG methylation analysis of mucilage using Matrix-Assisted Laser-Desorption Ionization Time-of-Flight Mass Spectra (MALDI-TOF) was used in Saez-Aguayo et al. (2013) to elegantly determine structural changes in HG. This technique involves digesting mucilage with polygalacturonases to hydrolyze HG into different oligogalacturonides with different degree of polymerization (DP). By detecting specific signals corresponding to unsaturated GalA oligomers, this method enabled the precise determination of changes in HG methylation (Saez-Aguayo et al., 2013). For detailed structural insights into RG-I and hemicelluloses, linkage analysis has also proven effective (Macquet et al., 2007b; Kong et al., 2013; Voiniciuc et al., 2015b; Fabrissin et al., 2019; Saez-Aguayo et al., 2021). This method can detect alterations in RG-I branching, as well as minor changes in xylan, galactan, arabinan and GCM structure structures within the SM layer (Macquet et al., 2007b; Kong et al., 2013; Voiniciuc et al., 2015b; Fabrissin et al., 2019; Saez-Aguayo et al., 2021).

Solid-state NMR (Nuclear Magnetic Resonance) has become a valuable method for investigating the structure and dynamics of cell wall polysaccharides (Pękala et al., 2023), including mucilage in *A. thaliana* seeds (Saez-Aguayo et al., 2014). Solid-state NMR allows for the analysis of cellulose, hemicellulose, and pectin networks in their natural hydrated state (Wang and Hong, 2016), which is crucial for understanding the polymer interactions in the cell wall matrix. For example, NMR techniques such as cross-polarization and magic-angle spinning (CP-MAS) help reveal how polysaccharide components like rhamnogalacturonan I (RG-I) in mucilage interact with cellulose (Pérez García et al., 2011; Lin et al., 2015), a primary structural component, contributing to the mucilage's functional properties. Research utilizing multidimensional solid-state NMR has provided insights into the structural nuances of pectic polysaccharides in Arabidopsis cell walls, which are central to mucilage's physical characteristics (Saez-Aguayo et al., 2014). Specific labeling techniques in solid-state NMR, like those with ^13^C isotopes, allow researchers to resolve fine structural details in pectins, enabling a better understanding of how modifications to these polysaccharides affect mucilage adhesiveness and hydration properties.

### 4.4 The traditional methods for separating pectin domains of mucilage

**Fig. 3:**
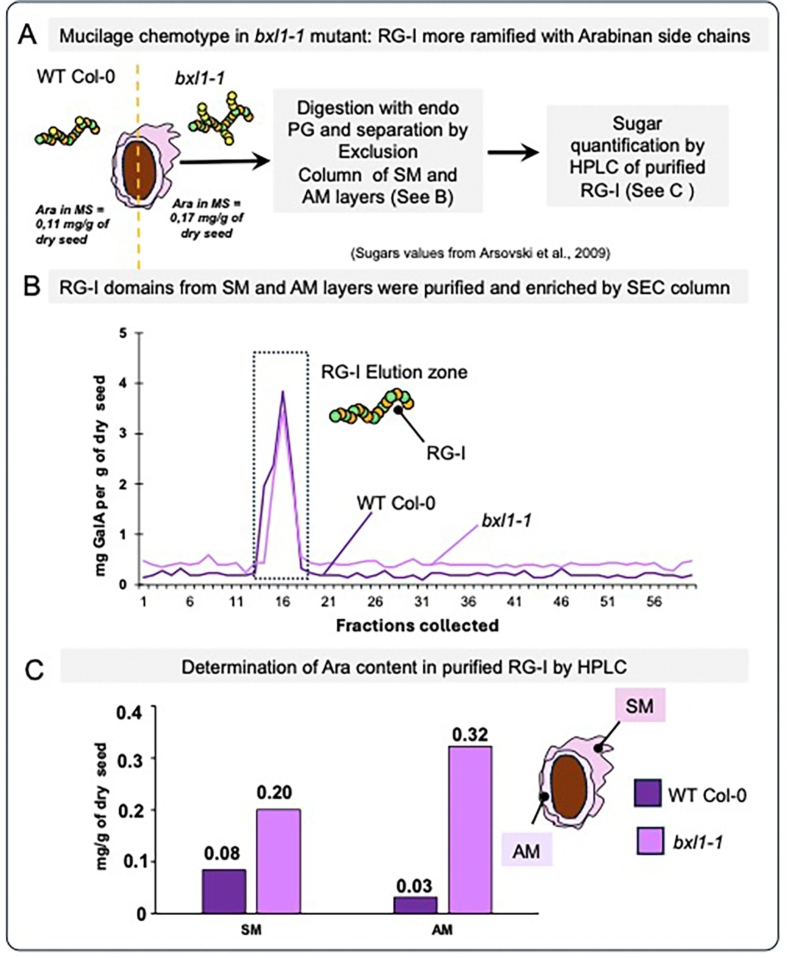
**Exploring old-fashioned methods of separating pectins by exclusion chromatography to purify mucilage RG-I.** A. Pipeline for the purification of RG-I mucilage from WT Col-0 seeds and *bxl1–1* mutant lines, which have been described to have more arabinan side chains (Arsovski et al., 2009; Williams et al., 2020). The *bxl1–1* mutant shows over 48 % of arabinose content in the SM compared to WT Col-0. B. Elution profile of pectins present in SM from WT Col-0 and *bxl1–1* mutant lines. The collected RG-I fractions are indicated, represented schematically as RG-I. C. RG-I from *bxl1–1* mutant mucilage contains higher arabinose levels. The separation of RG-I fractions was published in Sanhueza et al. (2024). Mucilage layers were extracted from mature seeds, digested with endoPG, and separated on a Bio-Gel P30 column. Fractions 14 to 19 were pooled, and sugar was analyzed by HPAEC-PAD following TFA hydrolysis.

Laboratories may encounter limitations due to restricted access to advanced technologies (e.g., HPSEC-MALL) and the limited availability of specific cell wall hydrolytic enzymes (e.g., RGH), which hinders comprehensive analysis of mucilage composition - particularly in studies of mutants with subtle phenotypes. To address these challenges, we applied a traditional size exclusion chromatography (SEC) method recently adapted for analyzing Arabidopsis seeds mucilage, based on protocols developed for pectin domains in Chilean papaya mucilage (Sanhueza et al., 2024a). Using 50 mg of dry seeds, the SM was extracted via water imbibition, while AM extraction involved sonication (Zhao et al., 2018). Mucilage layers were treated directly and loaded onto a Bio-Gel P-30 column for pectin domain separation as described in Sanhueza et al. (2024). Uronic acid content was assessed in fractions to identify those containing RG-I (Fig. 3B), which were then dried, reconstituted in water, hydrolyzed with TFA, and analyzed by HPAEC. To evaluate the method's robustness, we compared WT Col-0 mucilage with that of the *bxl1–1* mutant, which presents ramified RG-I (Arsovski et al., 2009; Williams et al., 2020). Consistent with Arsovski et al. (2009), we observed a 48 % increase in Ara content in *bxl1–1* GR-I from SM and AM (0.17 mg/g of dry seed in comparison to 0.11 mg of Ara /g per dry seed measured in WT-Col). We observed a similar increase in Ara content in the *bxl1–1* mutant in RG-I purified from SM and AM, validating this methodology as a reliable approach for analyzing distinct pectin domains. SEC enabled the isolation of RG-I but also allowed the isolation of oligogalacturonanides and the extremely low-abundant rhamnogalacturonan-II, providing material that can be collected for subsequent analyses (Sanhueza et al., 2024).

Another simple way to isolate RG-I involves ethanol precipitation. Following enzymatic digestion, acidic polysaccharides can be precipitated with a divalent (e.g., CaCl_2_) and at least 30 % ethanol (Smidsrød and Haug, 1967). This method allows the collection of larger fragments like RG-I (Macquet et al., 2007) via centrifugation, while leaving oligogalacturonides in solution. Additionally, RG-II can be eliminated by dialysis using a 12,000 MWCO (molecular weight cut-off) membrane, sufficient to exclude dimeric RG-II, estimated at 10 kDa (Chormova and Messenger, 2014).

## 5 How can mucilage analytical tools be effectively integrated to investigate the synthesis and modification of polysaccharides within the mucilage structure?

**Fig. 4:**
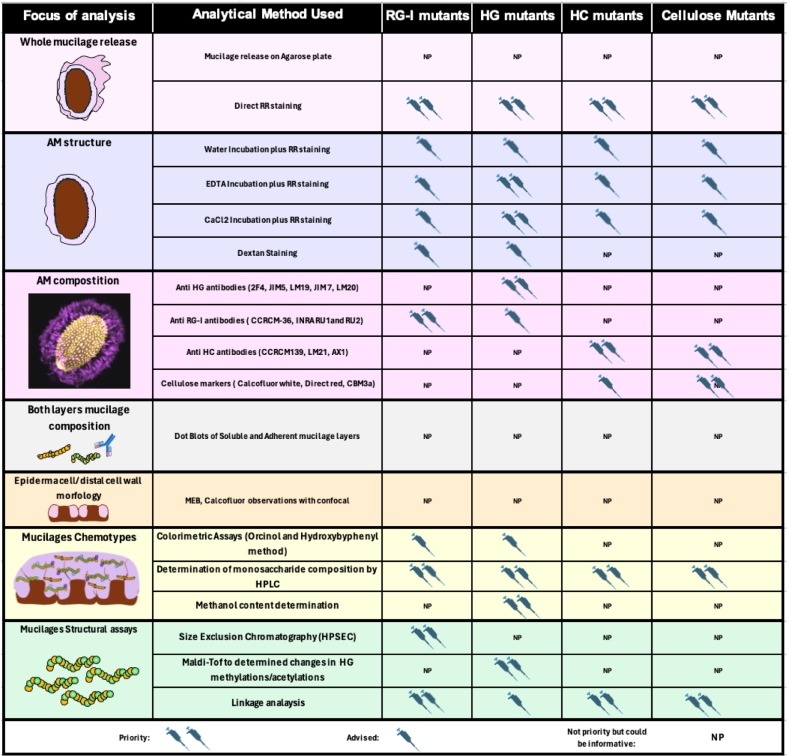
**“Muci map-guide” for phenotyping mucilage mutants.** This table was created to simplify mucilage mutant phenotyping. It summarizes techniques used for mucilage analysis, indicating the preferred methods recommended methods for mutant phenotyping with alterations in different pectin, hemicellulose, and cellulose polysaccharides.

In this section, we have organized mutants based on their roles in the synthesis and/or modification of RG-I, HG and hemicelluloses. We also compile the observed phenotypes using different techniques (Table 1, Table 2, Table 3). Based on this information, we outline the methods used and provide a roadmap to facilitate phenotyping mucilage mutants according to anticipated mucilage changes (Fig. 4).

**Table 2:** **Mucilage mutants altered in RG-I polymer and their corresponding phenotypic traits are outlined.** The table includes mutants with altered RG-I structure and associated phenotypes. The genetic functions are indicated as well as their main cytological and biochemical traits. n.r. (not reported).

Mutant	Protein function	Mucilage release (SM + AM)	RR (AM) phenotype	Immunolabeling with anti-CW antibodies	Biochemical changes and techniques used	Extras	Refs.
**RG-I backbone**							
urgt2 / urgt4 / urgt6 (*AT1G21070/ AT4G39390/ AT1G34020*)	UDP-Rha transporter (to Golgi lumen)	No obvious phenotype	No obvious phenotype	Less labeling with INRA-RU1 (RG-I).	Reduced Rha and GalA amounts (Colorimetric and HPAEC-PAD). Increased number of shorter RG-I molecules (HP-SEC). Increased amount of xylans (linkage analysis).	Reduced mucilage density with dextran FITC	Rautengarten et al. (2014) Saez-Aguayo et al. (2021)
*uuat1 (AT5G04160)*	UDP-GlcA and UDP-GalA transporter (to Golgi lumen)	No obvious phenotype	Less staining with EDTA	Decreased labeling with CCRC-M36 (RG-I) and LM6 (arabinan). Increased LM20 (high DM HG) labeling. Less Rutenium Red staining of AM after EDTA treatment	Reduced GalA, Rha and Xyl content (Colorimetric and HPAEC-PAD). Increased DM of HG.	n.r.	Saez-Aguayo et al. (2017)
*uuat3 (AT5G05820)*	Putative UDP-uronic acids transporter (to Golgi lumen)	No obvious phenotype	Higher staining with EDTA	Increase RG-I labeling with INRA-RU1	Reduced GalA, and Rha in SM and increased GalA in AM (Colorimetric and HPAEC-PAD).	n.r.	Parra-Rojas et al. (2019)
*mum4/rhm2* (AT1G53500)	Converts UDP-Glc to UDP-Rha	No mucilage release	n.r.	n.r.	Decreased Rha and uronic acid amounts (Colorimetric and HPAEC-PAD). Reduced RG-I amount and molecular weight (HP-SEC).	Flattened columela (MEB).	Western et al., 2001, Western et al., 2004 Usadel et al., (2004) Oka et al. (2007)
*rrt1 (AT5G15740)*	RG-I rhamnosyltransferase	n.r.	Lower halo/volume of extrusion in water	n.r.	Reduced Rha and GalA amounts (HPAEC-PAD).	Increased columella width (MEB)	Takenaka et al. (2018)
*gatl5 (AT1G02720)*	Putative galacturonosyltransferase	n.r.	Lower halo/volume of extrusion in water	n.r.	Reduced GalA and Rha amounts (HPAEC-PAD). Longer RG-I molecules (HP-SEC and linkage).	radial cell wall columella width affected (MEB)	Kong et al. (2013)
*muci70 / rggat1 (AT1G28240)*	RG-I galacturonosyltransferase	Lower halo	Lower halo/volume of extrusion in water	Less labeling or RG-I with CCRC-M36 and INRA-RU1, more labeling of xylan with CCRC- M139 and INRA-AX1	Decreased Rha and GalA content and an Increase in Xyl content (colorimetric and HPAEC-PAD). RG-I polymer with higher and length shorter of RG-I polymer (HP-SEC)	Collumelae shape less detectable (MEB), AM less dense (Dextran FITC)	Voiniciuc et al. (2018); Fabrissin et al., 2019
*cuaoa1 (AT1G31670)*	Cupper amine oxidase. Polyamine metabolism. Putative role in RG-I synthesis	No obvious phenotype	No obvious phenotype	No obvious phenotype	Reduced amounts of Rha and GalA in SM and slight increase of GalA in AM (Colorimetric and GC–MS)	n.r.	Fabrissin et al. (2019)
**RG-I side chains**							
*mum2 / bgal6* (AT5G63800)	β-galactosidase	no mucilage is released	no mucilage is released	no mucilage is released after alkali treatment to release mum2 mucilage, there is change in LM5 and JIM7 labeling in walls breakage. LM6 labeling is higher in the mum2 mutant.	Increased Gal content in AM. Reduced GalA and Rha content in SM and increased of both sugars in AM.	n.r.	Dean et al. (2007)Macquet et al. (2007b)
*ruby (AT1G19900)*	Galactose oxidase	Mucilage has a WT release	With water treatment the halo of AM is larger and more disheveled with stained dark particles	n.r.	Increase in Ara, Rha Gal and GalA content in mucilage extracted with Na_2_CO_3_ (HPAEC-PAD, colorimetric assay). Increase in RG-I branching (Linkage).	The presence of ruby particles corresponds to mucilage secretory cells detached from the seed coat	Šola and Dean (2019)
*rgp1 / rgp2* (AT3G02230/ AT5G15650)	UDP-arabinose mutase	Quick solubilization of mucilage layers.	Low AM staining with RR	n.r.	no data	n.r.	Rautengarten et al. (2011)
*uaft2 (AT5G11230)*	UDP-Arabinofuranose transporter (to Golgi lumen)	No visible phenotype	Less RR staining after EDTA treatment	Reduced INRA-RU1 (RG-I) labeling.	Reduced Ara content	n.r.	Parra-Rojas et al. (2019)
*bxl1 (AT5G49360)*	β-xylosidase / α-arabinofuranisidase	Patchy and delayed mucilage release	No differences with EDTA treatment	Increased LM6 (arabinan) labeling in MSC primary cell wall	Increased Ara Xyl and Fuc in SM (HPAEC-PAD) and reduced Xyl and Fuc content (HPAEC-PAD). More Ara ramification in SM (linkage)	RG-I more ramified with Arabinan side chains (AFM)	Arsovski et al. (2009); Williams et al. (2020)

**Table 3:** **Mucilage mutants altered in HGs domains and their corresponding phenotypic traits are outlined**. The Table includes mutants with altered HG synthesis and modifications, along with their associated phenotypes. Genetic functions are indicated, detailing their cytological and biochemical traits. n.r. (not reported).

Mutant /AGI number	Protein Function	Mucilage release (SM + AM)	RR Adherent Mucilage (AM) phenotype	Immunolabeling with anti-CW antibodies	Biochemical changes and techniques used	Extras	References
**HG synthesis**							
*gaut11 (AT1G18580)*	HG galactoturonosyltransferase	Patchy and delayed mucilage release with less staining	AM thinner and more stained	n.r.	Reduced Rha, GalA and Xyl. Increased Glc, Man and Gal (GC–MS). Changed in RG-I, HG, arabinan and galactan structure (linkage)	Reduced mucilage area with lower density (Dextran FITC). Flattened columella (MEB)	Caffall et al. (2009)Voiniciuc et al. (2018)
*gosamt1* / *2* /*3 (AT1G64650/ AT4G27720/ AT3G49310)*	Putative SAM transporters (to Golgi lumen)	Delayed mucilage release with less staining	Adherent mucilage has WT phenotype in water	Decrease JIM7 (high DM HG) AM labeling. Changes in S4B (cellulose) AM labeling. Decrease in JIM7 labeling and increase of LM30 in SM (Dot blots)	Reduced DM of HG (colorimetric assay). Reduced Rha, GalA and Xyl in SM and increased amounts of these sugars in AM (HPAEC-PAD).	Gaps in radial wall thickness and changes in distal wall length (Calcofluor)	Parra-Rojas et al. (2023)
*qua2 / tfa2 / tsd2 (AT1G78240)*	HG methyltransferase	Reduced mucilage area	n.r.	Changes in JIM5 (low DM HG) and JIM7 (high DM HG) labeling. Reduced S4B (cellulose) labeling	Reduced GalA. Decrease DM of HG. Reduced cellulose content	Increased columella area and decreased radial wall thickness (MEB)	Du et al. (2022)
*nks1/elmo4 (AT4G30996)*	Putative integral protein of a pectin synthesis protein complex. Phenocopy of *qua2*	n.r.	Reduced AM mucilage area	n.r.	Less GalA content in MS (HPAEC-PAD)	n.r.	Lathe et al. (2024)
**HG modification**							
*pme6/hms (AT1G23200)*	Pectin methylesterase	n.r.	Reduced AM mucilage area after water shaking	n.r.	Not differences in mucilage chemotype	The mucilage phenotype appears to result from alterations in embryo development	Levesque-Tremblay et al., 2015
*pme58 (AT5G49180)*	Pectin methylesterase	n.r.	Less AM staining after EDTA treatment	Changes in LM19 (Low DM HG) labeling after EDTA treatment	Increased DM of HG (colorimetric assay). Increased SM and reduced AM sugars in mucilage extracted with EDTA (HPAEC-PAD)	Decreased MSC surface area (MEB)	Turbant et al. (2016)
*pme31 (AT3G29090)*	Pectin methylesterase	Less mucilage staining and weak release phenotype	AM less stained with EDTA and CaCl_2_	n.r.	n.r.	n.r.	Zhang et al. (2023)
*pmei6 (AT2G47670)*	Pectin methylesterase inhibitor	No mucilage release	“Snake ski” residue attached to the seed after EDTA treatment and long imbibition in water and RR staining	Strong decrease in JIM5 (low DM HG) and JIM7 (high DM HG) labeling	Decreased DM of HG. Reduced sugar amounts in SM and increased in AM layers. GalA from HG is strongly reduced in both mucilage layers (HPAEC-PAD and colorimetric assays)	Delayed mucilage release.	Saez-Aguayo et al. (2013)
*pmei13 (AT4G15750)*	Pectin methylesterase inhibitor	n.r.	Halo of AM mucilage after water treatment is thinner	n.r.	n.r.	n.r.	Ding et al. (2021)
*pmei14 (AT1G56100)*	Pectin methylesterase inhibitor	n.r.	Bigger mucilage halo after NaOH treatment	Increased 2F4 (HG egg boxes) and LM19 (Low DM HG) labeling. Decrease in LM20 labeling (high DM HG)	Reduced methanol content (colorimetric assay).	Thickener cell wall (MEB). Increased calcium in mucilage	Shi et al. (2018); Ding et al., 2021; Allen et al. (2023)
*pmei15 (AT3G05741)*	Pectin methylesterase inhibitor	n.r.	Double mutant *pmei15erf4* have a subtle phenotype with a bigger halo	n.r.	Double mutant *pmei15erf4* have a subtle phenotype with a slight increase in the DM	n.r.	Ding et al. (2021)
*pmei18 (AT3G62820)*	Pectin methylesterase inhibitor	Less mucilage staining and weak release phenotype	AM less stained with EDTA and CaCl_2_	n.r.	No changes in GalA composition (Colorimetric assay)	n.r.	Zhang et al. (2023)
sbt1.7 / ara12 (AT5G67360)	Subtilase (Subtilisin-like serine proteases). Putative role in PME maturation	Delay in mucilage release	“Snake skin” residue attached to the seed after EDTA treatment a	No evident differences with JIM5 (low DM HG) and JIM7 (high DM HG) but abnormal breakage of the distal wall.	No changes in total amount of sugars (HPAEC-PAD). Less methanol content in MS and AM (colorimetric assay)	Delayed mucilage release. Mucilage extrusion is better using EDTA, but abnormal breakage of the distal wall.	Rautengarten et al. (2008)
fly1 / fly2 (AT4G28370/ AT2G20650)	RING E3 ubiquitin ligase. Putative role in PME recycling	Reduced mucilage release with capsule formation, particles with a disk structure shape	More AM labeling after EDTA treatment. CaCl_2_ increased a not mucilage release	Changes in JIM5 (low DM HG), JIM7 (high DM HG) and 2F4 (HG egg boxes) labeling	Less sugars in mucilage due to mucilage release. No changes in total amount of sugars (HPAEC-PAD)	In imbibed seeds cells are detached after imbibition (cryoSEM)	Voiniciuc et al. (2013); Kunieda et al. (2020)
*per36 (AT3G50990)*	Peroxidase	No mucilage release, phenocopy of pmei6	“Snake skin” residue attached to the seed after EDTA treatment and long imbibition in water and RR staining	n.r.	n.r.	n.r.	Kunieda et al., 2013
*tbl38 (AT1G29050)*	Atypical homogalacturonan acetylesterase	No mucilage release	No adherent mucilage	Changes in LM20 (High DM HG) labeling on developing seed section	No data	Reduction in LM20 labeling in the surface of MSCs	Dauphin et al. (2023)

### 5.1 Mutants affecting RG-I synthesis display a subtle phenotype that is challenging to discern using ruthenium red staining

Over the past two decades, various techniques have led to the characterization of approximately 90 genes involved in mucilage synthesis, deposition, and modification (Xu et al., 2023). Focusing on genes associated with RG-I synthesis and modification (Table 2) RR staining assays reveal that mutants in UDP- sugars conversions and Golgi nucleotide sugar transporters *mum4*, *uuat1*, *uuat3*, and *uaft2* exhibit mucilage release or non-adherent mucilage phenotypes (Table 2) (Saez-Aguayo et al., 2017; Parra-Rojas et al., 2019). These mutants also could exhibit methylation defects in their AM potentially explaining the RR staining phenotype observed following EDTA treatment (Table 2). This indicates that when RG-I content does not show a significant reduction in mucilage, cytological assays such as RR staining, are less effective in detecting mucilage phenotypes. In such cases, sugar quantification using colorimetric methods (e.g., *m*-hydroxybiphenyl and orcinol methods) and/or HPAEC-PAD analysis following TFA hydrolysis is recommended for identifying mucilage changes (Saez-Aguayo et al., 2017 and Saez-Aguayo et al., 2021). Immunolabeling with anti-RG-I and anti-galactan antibodies, like the LM5 antibody, also helps detect changes in RG-I distribution within the AM (Table2; Macquet et al., 2007a, Saez-Aguayo et al., 2021).

Further, determining RG-I structure through High-Performance SEC (HP-SEC) and linkage analysis is fundamental for identifying structural changes in RG-I (Fabrissin et al., 2019; Saez-Aguayo et al., 2021). If HP-SEC and/or linkage analyses are unavailable, traditional SEC, as used for *bxl-1*, may be employed. Also, dot-blot analysis using antibodies that target minor mucilage components, such as LM6 (anti-arabinan) and LM5 (anti-galactan), which recognize RG-I side chains, can effectively detect structural changes in fractions collected through SECs and/or extracted mucilage (Parra-Rojas et al., 2023; Sanhueza et al., 2024).

### 5.2 Mutants with alterations in HG exhibit a wide range of phenotypes, can be detected using Ruthenium Red staining

Genes involved in the synthesis and modification of HG, including methylation and acetylation processes, have been extensively characterized, largely due to mucilage studies (Table 2). A range of mucilage phenotypes has been identified, from strong phenotypes, such as *pmei6, sbt1.7* or *prx36,* which do not release their mucilage, to more discrete phenotypes like the delayed mucilage release observed in *gosamts* mutants (Saez-Aguayo et al., 2013; Kunieda et al., 2013; Parra-Rojas et al., 2023; Rautengarten et al., 2008). Despite the diversity, mutants affecting HG typically exhibit alteration in the AM structure, which is easily observable by RR staining following seed imbibition in water, EDTA, and CaCl_2_ (Table 2). Therefore, assessing AM structure is often the first step in characterizing HG mutants.

Characterizing HG mutant can be challenging, as HG represents only a small fraction of mucilage components, and mutations in HG metabolism sometimes result in minimal changes to the HG methylation pattern. To detect changes more thoroughly, immunolabeling using a panel of anti-HG antibodies (Table S1 X; JIM5, JIM7, LM20, LM19, and 2F4) has proven effective, revealing both significant and subtle changes in HG methylesterification patterns. For these mutants, it is advisable to quantify methanol and GalA released from pectin to calculate the DM (Fig. 4). Before doing so, however, it is essential to separate HG domains by precipitation with ethanol after RG-I hydrolysis or by using SEC, as shown by Saez-Aguayo et al. (2013) and Sanhueza et al. (2024). This ensures accurate DM assessment by excluding GalA from the RG-I backbone, which would otherwise underestimate the true DM of HG in mucilage.

### 5.3 The cellulose and hemicellulose: mutants exhibiting hypersolubilization of their mucilage

Due to the low content of hemicelluloses in mucilage, mutants typically exhibit mild mucilage phenotypes, with total mucilage release resembling that of WT lines (Table 4). Interestingly, for AM all mutants except *muci10* exhibit a reduced halo or changes in the AM staining when incubated in water, but not in EDTA or CaCl_2_ (Table 4). Generally, hemicellulose mutants have a more soluble mucilage layer and reduced AM, as hemicelluloses contribute to AM adherence (Table 4). Immunolabeling provides more detailed insights for these mutants, with positive labeling seen using AX1, CCRC-M139, LM21, and CBM3a antibodies. Additionally, oriented crystalline cellulose can be detected based on light birefringence, while changes in cellulose organization and structure are generally assessed using calcofluor, pontamine, and CBM staining (Mendu et al., 2011). All mutants display changes not only in glucose content but also in other sugars, highlighting the importance of performing sugar determination using HPAEC-PAD or GC–MS.

**Table 4:** **Mucilage mutants altered in cellulose and hemicellulose polymer and their corresponding phenotypic traits are outlined.** The table includes mutants with altered hemicellulose and cellulose structure and associated phenotypes. Genetic functions are indicated, detailing their main cytological and biochemical traits. n.r. (not reported).

Hemicellulose mutants							
Mutant /AGI number	Protein function	Mucilage release (SM + AM)	RR (AM) phenotype	Immunolabeling with anti-CW antibodies	Biochemical changes and techniques used	Extras	Refs.
**Xylan synthesis**							
*irx14*	Putative Xylan β-1,4-xylosyltransferase (backbone)	n.r.	Reduced AM due to loss of adherence.	Reduced labeling with CCRC-M139 (xylan) and LM11 (highly branched xylan)	Reduced Xyl content. Increased Man content in SM and Ara in AM.	Reduced crystalline cellulose and structure.	Voiniciuc et al. (2015b) Hu et al. (2016b)
*muci21 / mum5*	Putative Xylan β-1,2-xylosyltransferase (side chains)	n.r.	Reduced AM due to loss of adherence.	Different CCRC-M36 (RG-I), CCRC-M139 (xylan) and AX1 (xylan) distribution	Reduced Xyl content. Increased GalA in SM and reduced in AM	Impaired cellulose structure by S4B labeling.	Western et al. (2001) Voiniciuc et al. (2015b) Ralet et al. (2016)
*irx7*	Putative xylan xylosyltransferase	n.r.	n.r.	Reduced labeling with CCRC-M139 (xylan) and LM11 (highly branched xylan).	Reduced Xyl content. Increased Rha and GalA in SM and decreased in AM	Reduced CBM3a (crystalline cellulose) labeling. Cell adhesion between MSCs is affected.	Hu et al. (2016a)
**Galactoglucomannan synthesis**							
*vtc1*	GDP-Mannose pyrophosphorilase	Slight reduction in mucilage area.	n.r.	Reduction in LM19 (unesterified HG) and calcofluor (b-glucans) labeling.	Reduced Man in AM.	n.r.	Nishigaki et al. (2021)
*csla2*	GGM mannosyltransferase	n.r.	Reduced AM area and lower density.	Changes in LM21 (heteromannans) labeling distribution. Changes in distribution of CCRC-M14 (unsubstituted RG-I), JIM5 (low DM HG) and JIM7 (high DM HG) labeling	Reduced Man and Glc in SM and after 2N NaOH extraction.	Reduced crystalline cellulose with altered distribution	Yu et al. (2014), Yu et al. (2018)
*muci10* / *magt1*	GGM galactosyltransferase	Reduced mucilage area and density	AM is less adherent.	n.r.	Reduced Gal, Glc and Man.	Reduced S4B (cellulose) and CBM3a (crystalline cellulose) labeling distribution.	Voiniciuc et al. (2015c)
**Cellulose mutants**							
**Mutant /AGI number**	**Protein function**	**Mucilage release (SM + AM)**	**RR (AM) phenotype**	**Immunolabeling with anti-CW antibodies**	**Biochemical changes and techniques used**	**Extras**	**References**
**Cellulose synthesis**							
*cesa5 / mum3*	Cellulose synthase	More soluble mucilage du to less AM adherence	Reduced mucilage area by loss of AM adherence.	Abnormal distribution of JIM5 (low DM HG), JIM7 (high DM HG), CCRC-M36 (unsubstituted RG-I) labeling	Sugars more easily extractable, therefore, reduced sugar quantities in AM and increases in SM	Decrease in radial wall thickness. Loss of organization of cellulosic rays and cellulose distribution using calcofluor (b-glucans), S4B (cellulose), CBM28 (amorphous cellulose) and CBM3a (crystalline cellulose). A	Western et al. (2001)Harpaz-Saad et al. (2011) Mendu et al. (2011)Sullivan et al. (2011)Griffiths et al. (2015)
*cesa3* / *irx1*	Cellulose synthase	Reduced mucilage area.	n.r.	Changes in CCRC-M36 (unsubstituted RG-I) and JIM5 (low DM HG) labeling	Reduced crystalline cellulose. Less Rha in SM.	Reduced cellulosic rays and abnormal CBM28 (amorphous cellulose) and CBM3a (crystalline cellulose) distribution.	Griffiths et al. (2015)
*cobl2*	GPI-anchored COBRA-LIKE protein. Synthesis and assembly of crystalline cellulose	More soluble mucilage du to less AM adherence	Reduced mucilage area by loss of AM adherence.	n.r.	Reduced crystalline cellulose. Reduced sugars in AM and increases in SM.	Decrease in crystalline cellulose deposition and distribution by calcofluor (b-glucans) and S4B (cellulose)	Ben-Tov et al. (2015) Ben-Tov et al. (2018)

Linkage analysis is crucial for detecting subtle changes in HC structure in mucilage. If this is not feasible, PACE analysis of GGM extracted from mucilage, as demonstrated by Nishigaki et al. (2021), can be used for HG structure analysis, highlighting the need to develop traditional techniques for studying HC in mucilage.

Given the close interaction between cellulose and HC, their mutants often exhibit similar phenotypes. The *cesA5* mutant exhibits a pronounced phenotype, characterized by high solubility of nearly all AM. This results from impared adhesion of RG-I to beta-glucan chains of cellulose, leading to an increased SM layer. It has been shown that CESA5 works in conjunction with CESA3 to form the AM cellulose matrix, alongside the GPI-anchored COBRA-LIKE protein (COBL2). Mutants of these genes exhibit similar phenotypes, with varying degrees of reduced AM and increased SM solubilization, which can be easily identified using RR staining. CESA5, along with CESA2 and CESA9, contributes to cellulose formation in the secondary cell wall of the radial and distal walls of epidermal cells. Triple *cesa5x2x9* mutants exhibit changes in cellulose organization in AM due to the mutation of CESA5, resulting in alterations to the radial thickness of epidermal cells and the morphology of the distal cell wall.

## 6 Conclusions

In this review, we summarized the importance of developing simplified models to study cell wall metabolism in plants, given the inherent complexity and current limitations of such research. Mucilage serves as an excellent model system for investigating the synthesis and modification of primary cell wall components, allowing the identification of key factors involved in the synthesis of HG, RG-I, and HC. We discussed various techniques used to phenotype mucilage mutants, discussing their advantages and limitations, and proposed a workflow for comprehensive mutant characterization.

Despite substantial progress over the past two decades, the metabolism of pectin acetylation remains completely unknown, and our understanding of RG-II synthesis and modification is still limited. This highlights the urgent need for the development of additional simplified systems to advance our knowledge. Additionally, we emphasize the importance of more easily accessible techniques to facilitate cell wall research, particularly in laboratories with limited funding. Finally, the proposed pipeline aims to streamline the study of mucilage mutants, enhancing our understanding of plant cell wall biology.

## Author contribution

SSA - Design the research; SSA, DS and AL-G, write the article; DS, VJ, BG and AL-G create the tables; SS-A, VJ and BG created Figures; DS, realized the experiments; AGL, AM and AGR revised the manuscript.

During the preparation of this work, the author(s) used ChatGPT AI to correct the language. After using this tool/service, the author(s) reviewed and edited the content as needed and assume full responsibility for the publication's content.

## Fundings

The work has received financial support from ANID-Anillo ACT210025 project, Fondecyt 1201467 (to SS-A), ECOS 210032 (to SS-A), MNSAP (to SS-A).

## CRediT authorship contribution statement

**Susana Saez-Aguayo:** Writing – review & editing, Writing – original draft, Supervision, Methodology, Investigation, Funding acquisition, Data curation, Conceptualization. **Dayan Sanhueza:** Writing – review & editing, Writing – original draft, Methodology, Formal analysis, Data curation, Conceptualization. **Vicente Jara:** Writing – original draft. **Benjamin Galleguillos:** Writing – original draft, Conceptualization. **Alfonso Gonzalo de la Rubia:** Writing – original draft, Conceptualization. **Asier Largo-Gosens:** Writing – original draft, Conceptualization. **Adrian Moreno:** Writing – review & editing.

## Declaration of competing interest

The authors declare that they have no known competing financial interests or personal relationships that could have appeared to influence the work reported in this paper.

## Data Availability

No data was used for the research described in the article.

## Appendix A Supplementary data

Supplementary material Image 1
