# Supplementary material for: Mucilicious methods: Navigating the tools developed to Arabidopsis Seed Coat Mucilage analysis
Source: Cell Surf. 2024 Dec 11;13:100134. doi: 10.1016/j.tcsw.2024.100134 (PMC11696855; doi:10.1016/j.tcsw.2024.100134)
Supplement: Supplementary file 1 — Supplementary material [file mmc1.docx]

**References**:

Clausen, M.H., Willats, W.G.T., Knox, J.P. (2003). Synthetic methyl hexagalacturonate hapten inhibitors of anti-homogalacturonan monoclonal antibodies LM7, JIM5 and JIM7. Carbohydr.Res. 338:1797-1800, 2003. doi:10.1016/S0008-6215(03)00272-6

Guillon, F., Tranquet, O., Quillien, L., Utille, J.P., Ortiz, J.J.O., Saulnier, L. (2004). Generation of polyclonal and monoclonal antibodies against arabinoxylans and their use for immunocytochemical location of arabinoxylans in cell walls of endosperm of wheat. J.Cereal Sci. 40:167-182. doi:10.1016/j.jcs.2004.06.004

Jones, L., Seymour, G.B., Knox, J.P. (1997). Localization of pectic galactan in tomato cell walls using a monoclonal antibody specific to (1->4)-beta-D-galactan. Plant Physiol. 113:1405-1412. doi:10.1104/pp.113.4.1405

Knox, J.P., Linstead, P.J., King, J., Cooper, C., Roberts, K. (1990). Pectin esterification is spatially regulated both within cell walls and between developing tissues of root apices. Planta 181:512-521. doi.org/10.1007/BF00193004

Liners, F., Van Cutsem, P. (1992). Distribution of pectic polysaccharide throughout walls of suspension-cultured carrot cells. An immunocytochemical study. Protoplasma 170:10-21. doi:10.1007/BF01384453

Liners, F., Letesson, J.-J., Didembourg, C., Van Cutsem, P. (1989). Monoclonal antibodies against pectin. Recognition of a conformation induced by calcium. Plant Physiol. 91:1419-1424. doi:10.1104/pp.91.4.1419

Liners, F., Thibault, J.-F., Van Cutsem, P. (1992). Influence of the degree of polymerization of oligogalacturonates and of esterification pattern of pecin on their recognition by monoclonal antibodies. Plant Physiol. 99:1099-1104. doi:10.1104/pp.99.3.1099

McCartney, L., Marcus, S.E., Knox, J.P. (2005). Monoclonal antibodies to plant cell wall xylans and arabinoxylans. J Histochem Cytochem. 53: 543-546. doi:10.1369/jhc.4B6578.2005

Moller, I., Marcus, S.E., Haeger, A., Verhertbruggen, Y., Verhoef, R., Schols, H., Ulvskov, P., Mikkelsen, J.D., Knox, J.P., Willats, W.G.T. (2008). High-throughput screening of monoclonal antibodies against plant cell wall glycans by hierarchical clustering of their carbohydrate microarray binding profiles. Glycoconj J. 25: 37‐48. doi.org/10.1007/s10719-007-9059-7

Verhertbruggen, Y., Marcus, S.E., Haeger, A., Ordaz-Ortiz, J.J., Knox, J.P. (2009). An extended set of monoclonal antibodies to pectic homogalacturonan. Carbohydr.Res. 344:1858-1862. doi:10.1016/j.carres.2008.11.010

Willats, W.G.T., Marcus, S.E., Knox, J.P. (1998). Generation of a monoclonal antibody specific to (1->5)-alpha-L-arabinan. Carbohydr.Res. 308:149-152. doi:10.1016/S0008-6215(98)00070-6

Willats, W.G.T., Limberg, G., Buchholt, H.C., Van Alebeek, G.-J., Benen, J., Christensen, T.M.I.E., Visser, J., Voragen, A., Mikkelsen, J.D., Knox, J.P. (2000). Analysis of pectic epitopes recognised by hybridoma and phage display monoclonal antibodies using defined oligosaccharides, polysaccharides, and enzymatic degradation. Carbohydr.Res. 327:309-320, 2000. doi:10.1016/S0008-6215(00)00039-2
